# Breaking the ice − simplified freeze-fracture of parasitic protists: a cost-effective approach without highly specialised equipment

**DOI:** 10.1590/0074-02760250060

**Published:** 2025-12-15

**Authors:** Marcia Attias, Luis Otávio da Silva Pacheco, Sharmila Fiama das Neves Ortiz, Raphael Verdan, Kildare Miranda, Ana Paula Gadelha, Wanderley de Souza, Marlene Benchimol

**Affiliations:** 1 Universidade Federal do Rio de Janeiro, Centro de Pesquisa em Medicina de Precisão, Instituto de Biofísica Carlos Chagas Filho, Laboratório de Ultraestrutura Celular Hertha Meyer, Rio de Janeiro, RJ, Brasil; 2 Universidade Federal do Rio de Janeiro, Centro Nacional de Biologia Estrutural e Bioimagem, Instituto Nacional de Ciência e Tecnologia em Biologia Estrutural e Bioimagem, Rio de Janeiro, RJ, Brasil; 3 Universidade do Estado do Amazonas, Centro Multiusuário para Análise de Fenômenos Biomédicos, Manaus, AM, Brasil; 4 Instituto Nacional de Metrologia, Qualidade e Tecnologia, Diretoria de Metrologia Científica, Laboratório de Biologia de Células Eucariontes, Duque de Caxias, RJ, Brasil; 5 Universidade do Grande Rio, Duque de Caxias, RJ, Brasil

**Keywords:** freeze-fracture, deep-etching, Toxoplasma gondii, Trichomonas vaginalis, Trypanosoma cruzi, Giardia intestinalis, osmium maceration

## Abstract

**BACKGROUND:**

We present a simplified freeze-fracture method to reveal the internal structure of protozoan parasites and their host cells, without the need for costly transmission electron microscopy (TEM) equipment. In a traditional procedure, the four key steps in making a freeze-fracture replica are (1) freezing, (2) fracturing, (3) replication, and (4) replica cleaning. In conventional protocols, visualisation is performed using a TEM.

**OBJECTIVES:**

This study aimed to adapt the traditional freeze-fracture protocol to a more accessible method, eliminating the need for vacuum equipment and TEM, while preserving the capacity to visualise cellular structures.

**METHODS:**

In our adapted method, vacuum equipment was not required for sample fracturing, shadowing, or replica preparation. Cells were fixed with glutaraldehyde, embedded in a gelatin-chitosan matrix, cryoprotected with dimethyl sulfoxide (DMSO), and subsequently frozen in Freon, followed by immersion in liquid nitrogen. We manually break the cells with a previously frozen razor blade and macerate them with osmium tetroxide (OsO_4_). The samples are visualised using scanning electron microscopy (SEM).

**FINDINGS:**

We present various structures of *Toxoplasma gondii*, *Trypanosoma cruzi*, *Trichomonas vaginalis*, *Giardia intestinalis*, and cultured cells observed using our adapted freeze-fracture method.

**MAIN CONCLUSIONS:**

This is a rapid and low-cost technique that reveals cell structures comparable to those observed using traditional freeze-fracture methods, although with reduced resolution.

Freeze-fracture is a crucial tool for cell studies under electron microscopy, pioneered by Moor and Mühlethaler.^1^ In addition, when vacuum sublimation (etching) is used, it reveals additional cell details, and the method is referred to as freeze-etching. From this time, several authors demonstrated that it was possible to use living cells and tissues, fix them with glutaraldehyde, followed by cryoprotection with glycerol, freeze the sample at low temperatures, and fracture it in equipment under a vacuum using a frozen sharp steel blade.^2^ After fracturing, the exposed surface is shadowed with a thin platinum-carbon layer. Organic material is then removed using corrosive agents, such as acids or sodium hypochlorite. A copy of the fractured surface replica is obtained, referred to as a replica, and examined using transmission electron microscopy (TEM). It was demonstrated that membranes are fractured along different cleavage planes, revealing internal cell structures and organelles. The images obtained revealed a new vision of the cells’ morphology and substructure; it was possible to visualise particles in the size range of large proteins on fractured membranes.

It is well known that several proposals for models of biological membrane structure were made in the 1960s and early 1970s. It has been accepted that membranes split during freeze-fracture, and the spherical structures appearing in freeze-fractured biological membranes are generally considered to be proteins inserted into the bilayer. The freeze-fracture technique contributed to the elaboration of the classic fluid-mosaic model of the cell membrane, which remains prevalent today.

The freeze-fracture and freeze-etch methods have been widely used in various circumstances and applications.^2^ For instance, they provided knowledge concerning the molecular organisation of cell junctional complexes and all organellar membranes, including mitochondrial and chloroplast membranes.^3^ ^,^ ^4^

Fukudome and Tanaka^5^ described a technique for examining the internal structures of cultured free cells (ascitic hepatoma cells, AH100B, and African green monkey kidney, BSC-1) using scanning electron microscopy (SEM). Their method involved embedding the samples in a gelatin and chitosan mixture, followed by maceration in OsO_4_. They reported that this polysaccharide is unaffected by osmium maceration, resulting in a fracture face that appears featureless at low magnification and does not introduce significant artefacts. The procedure integrates chemical fixation, cryoprotection, freezing, manual fracturing, and maceration using low-concentration OsO_4_, which partially removes the cytoplasmic matrix while preserving membranes, organelles, and other cellular structures. Their protocol includes maceration for three to five days, depending on the cell type, followed by refixation with 1% OsO_4_ and treatment with 2% tannic acid.^6^ Finally, the samples are dehydrated, subjected to critical-point drying, and coated with platinum before SEM observation.

A simplified freeze-fracture technique combined with OsO_4_ maceration can provide valuable insights into the morphological and structural organisation of protozoan parasites. This enhanced visualisation is crucial for understanding pathogenic mechanisms and interactions with the host cells. Here, we explored and adapted methods that do not require specialised equipment, facilitating more rapid and cost-effective studies and thereby contributing to the development of a solid foundation in the cell biology of parasitic protists.

To ensure reproducibility, all experiments were independently performed six times. Furthermore, due to the inherent fragmentation of the sample preparation process, multiple fragments from each sample were analysed, thereby increasing the robustness and representativeness of the data.

## MATERIALS AND METHODS

A minimum of six experiments were performed for each cell analysed here, and a minimum of 30 images were obtained from each experiment. All cell lines complied with the ethical standards established at this University.

*Toxoplasma gondii protocol* - Rhesus monkey kidney epithelial cells (LLCMK2) were plated in Falcon culture flasks and infected with *T. gondii* [Rhesus (RH) strain] (MOI 1:10). After 48 h of infections, the cells were trypsinised to release them from the flask, gently centrifuged in a clinical centrifuge (2000x g), and immediately fixed with 2.5% glutaraldehyde in 0.1 M cacodylate buffer for 2 h, rinsed in the same buffer and post-fixed in 1% OsO_4_ in the same buffer for 1 h, rinsed in the same buffer three times, 10 min each. Subsequently, the pellet was resuspended in a small buffer volume and mixed 1:1 with 10% gelatin and 2% chitosan, as described in Fukudome and Tanaka.^5^ The sample was cut into 2 mm-thick slices and refixed in 2.5% glutaraldehyde in cacodylate buffer for 2 h, rinsed, and cryoprotected with dimethyl sulfoxide (DMSO) 25% and 50% for 30 min in each concentration. The fragments were then immersed in liquid nitrogen, placed over an N_2_-cooled brass plate, and cleaved with a razor blade that had been pre-cooled in liquid nitrogen. Fractured fragments were thawed and rinsed in distilled water to remove DMSO and macerated for 24-48 h in 0.1% OsO_4_ in water. The fragments were rinsed again in distilled water, dehydrated in ethanol, critical-point dried, mounted on aluminum stubs, sputtered with gold, and observed in a ZEISS Auriga 40 Field Emission SEM operating at 2 kV.

*Protocol for RAW 264.7 cells infected with Trypanosoma cruzi strain Y* - This study used the RAW 264.7 macrophage cell line from mice (ATCC, Rockville, MD, USA). The cells were maintained in a complete DMEM medium (DMEM-c) at 37ºC in a humidified incubator with 95% air and 5% CO_2_. The cells were allowed to interact with *T. cruzi* epimastigotes strain Y at a ratio of 1:10. After 24 h of infection, the cells were trypsinised and placed in a 15 mL Falcon tube for separation from the culture medium. The cells were gently centrifuged and fixed with 2% glutaraldehyde plus 0.5% formaldehyde in 0.1 M cacodylate buffer for 2 h. Subsequently, they were washed in the same buffer and post-fixed in 1% OsO_4_ for 1 h, followed by three washes in the same buffer for 10 min each. The pellet was resuspended in a mixture of 10% gelatin and 2% chitosan, and drops of this new mixture were prepared. This mixture was refrigerated for 20 min to polymerise the drops, which were then submerged in 2.5% glutaraldehyde in water for 30 min to reinforce the firmness of the drops. Next, the drops were washed in the fixative buffer and underwent infiltration with 25% and 50% DMSO for 30 min each. The drops were pre-frozen in cooled Freon 22 and immediately transferred to liquid nitrogen. The samples were positioned on a platform submerged in liquid nitrogen and fractured with a razor blade. The fragments were washed again in distilled water to remove excess DMSO. The fragments were then subjected to maceration in 0.1% OsO_4_ solution in water for 96 h, with the solution changed every 24 h. The fragments were then washed three times in water for 10 min each and dehydrated in a graded ethanol series (30%, 50%, 70%, 90%, and 100%) for 10 min each. Subsequently, the samples were dried using the critical point method (Leica CPD 300, Germany). After drying, the fractured surfaces of the fragments were placed face-up on a carbon tape-lined stub. Next, to complete the processing, the fragments on the support were coated with a 10 nm thick conductive layer of platinum. These samples were observed using a field-emission SEM (Auriga) with a working distance of 10 mm. Some fragments were not coated with conductive metal and were observed with the Orion NanoFab high-resolution Helium ion microscope. Other tentative protocols are shown in Table.

**TABLE:** Summary table of the tested protocols and the corresponding scanning electron/ion microscope (SEM) models used

Sample		Cryoprotector	Maceration	Coating	SEM model
1	LLC-MK2 + *Toxoplasma gondii*	25%-50% DMSO	24-48 h	Gold	HR-AURIGA 40 ZEISS
2	LLC-MK2 + *Trypanosoma cruzi* trypomastigotes	25%-50% DMSO	24-48 h	No coating	EVO 10 ZEISS
				Gold	
				Platinum	
3	LLC-MK2 + *T. cruzi* trypomastigotes	25%-50% DMSO	No maceration	Platinum	EVO 10 ZEISS, HR-AURIGA 40 ZEISS, ION-ORION NANOFAB ZEISS
4	RAW + *T. cruzi* epimastigotes	25%-50% DMSO	72-144 h	No coating	EVO 10 ZEISS, ION-ORION NANOFAB ZEISS
				Platinum	
5	*T. cruzi* epimastigotes	25%-50% DMSO	No maceration	No coating	EVO 10 ZEISS, HR-AURIGA 40 ZEISS, ION-ORION NANOFAB ZEISS
				Gold	
6	*Giardia intestinalis*	25%-50% DMSO	7 days	Platinum	QUATTRO S THERMO FISHER, HR-AURIGA 40 ZEISS
	*Trichomonas vaginalis*				

DMSO: dimethyl sulfoxide; LLC-MK2: Rhesus monkey kidney epithelial cells; RAW: macrophage cell line.

*Trichomonas vaginalis and Giardia intestinalis protocol* - The strain JT of *T. vaginalis* was isolated from a patient attending the Federal University of Rio de Janeiro Hospital (Rio de Janeiro, RJ, Brazil). The cells were maintained at 37ºC and subcultured daily in trypticase yeast-extract maltose medium (TYM),^7^ supplemented with 10% foetal bovine serum (FBS) (Gibco, USA). The freeze-fracture protocol of *T. vaginalis* for SEM observation was modified from the protocol of Fukudome and Tanaka.^5^ After detaching the cells from the tube wall by leaving them on ice for 15 min, they were centrifuged and washed twice with phosphate-buffered saline (PBS), pH 7.2. The cells were fixed in 0.5% formaldehyde and 2% glutaraldehyde in 0.1 M sodium cacodylate buffer, pH 7.2, for 1 h at room temperature (RT). After washing off the fixative, the cells were post-fixed for 40 min with 1% OsO_4_ in 0.1 M cacodylate buffer containing 5 mM CaCl_2_ and 0.8% potassium ferricyanide. Subsequently, the samples were mixed with an equal volume of a solution containing 10% gelatin in water and 5% chitosan diluted in 0.25% acetic acid. 50-100 μL drops were formed on a piece of parafilm and placed in the refrigerator for 20 min to harden. The solidified drops were refixed with 2.5% glutaraldehyde in 0.1 M sodium cacodylate buffer, pH 7.2, for 30 min. After washing three times with the same buffer, the samples were cryoprotected with 25% and 50% DMSO solutions in water for 30 min each. The samples were frozen in Freon and then in liquid nitrogen, and fracture was performed using a pre-cooled razor blade. After fracturing, the fragments were washed with water, and maceration was performed.

The maceration process involved daily changes of a 0.1% OsO_4_ solution in water for seven days at RT. After this process, the samples were washed three times in water and dehydrated in increasing ethanol concentrations, 15 min in each concentration. Then, the samples were dried using the critical point method (Leica CPD 300, Germany) and mounted on stubs with the fractured face facing upwards. Finally, they were coated with 10 nm of platinum (Quorum Technologies, United Kingdom) and observed on an Auriga 40 SEM (Zeiss, Germany) or Quattro S (Thermo Fisher, USA).

Trophozoites of the WB strain of *G. intestinalis* were cultivated axenically in TYI-S-33 medium, pH 7.2, supplemented with 0.1% bovine bile and 10% FBS.^8^ Cultures were maintained in 15 mL tubes at 37ºC for 48 h. Tubes containing cells in the log phase were used in the experiment. The freeze-fracture protocol for *G. intestinalis* was the same as that for *T. vaginalis* mentioned above.

## RESULTS AND DISCUSSION

The freeze-fracture methods have been used in various cells and tissues, enabling significant advances in the study of cell organisation. Obtaining replicas made from heavy metals and carbon enabled notable advances in understanding cellular ultrastructure, including different cell types and protozoa of various origins and pathogenicities, information that was previously inaccessible with conventional sample preparation techniques for studying cellular architecture. However, this methodology is expensive. It requires high-cost, sophisticated equipment and several litres of liquid nitrogen to function effectively in a single experiment. Furthermore, someone with expertise in handling the equipment, as well as obtaining and cleaning the replicas, is essential. Additionally, the equipment operates only in a high vacuum, which necessitates near-perfect operation.

OsO_4_ is a symmetric, nonpolar molecule with a high molecular weight (254.23 g/mol), preventing coagulation and lipoprotein rupture.^9^ This characteristic provides contrast and electron density to the phospholipids in the plasma membrane, binding to unsaturated fatty acids. During OsO_4_ fixation, initial gelation occurs, partially rendering proteins insoluble through denaturation and consuming many cellular components. This process may be followed by further protein denaturation, resulting in soluble products and, ultimately, final hydrolysis of certain end products that can be washed out of the cell after prolonged fixation.^10^

Here, we adapted and introduced a practical, cost-effective freeze-fracture technique with osmium maceration. This method allows us to observe a variety of cells and protist parasites, including *T. gondii*, *T. cruzi*, *G. intestinalis*, and *T. vaginalis* [Table, Supplementary data (Figure)]. The samples were observed in a routine SEM. In contrast, others were metal-uncoated and observed at high resolution using a scanning ion microscope Orion NanoFab with a working distance of 8.5 mm. This instrument enables high-resolution imaging with high surface sensitivity and a depth of field 5-10 times higher than that of a conventional SEM, making it a promising tool for future research.

*Toxoplasma gondii* - *T. gondii* is a cosmopolitan parasite with a high prevalence worldwide.^11^ ^,^ ^12^ ^,^ ^13^ It is the causative agent of toxoplasmosis, and although the infection is usually asymptomatic, *T. gondii* is a serious pathogen when it affects immunocompromised patients and congenitally infected newborns.^14^ In the acute stage of the infection, any nucleated cell of the host can be actively invaded by tachyzoites, which remain in a parasitophorous vacuole (PV).^15^ In this process, the parasites divide through a special mechanism known as endodyogeny.^16^ ^,^ ^17^ Consecutive divisions enlarge the PV that contains parasites arranged in a three-dimensional array known as a rosette. After a few days, the vacuole ruptures, releasing many infective stages into the intercellular space.

**Fig. 1:**
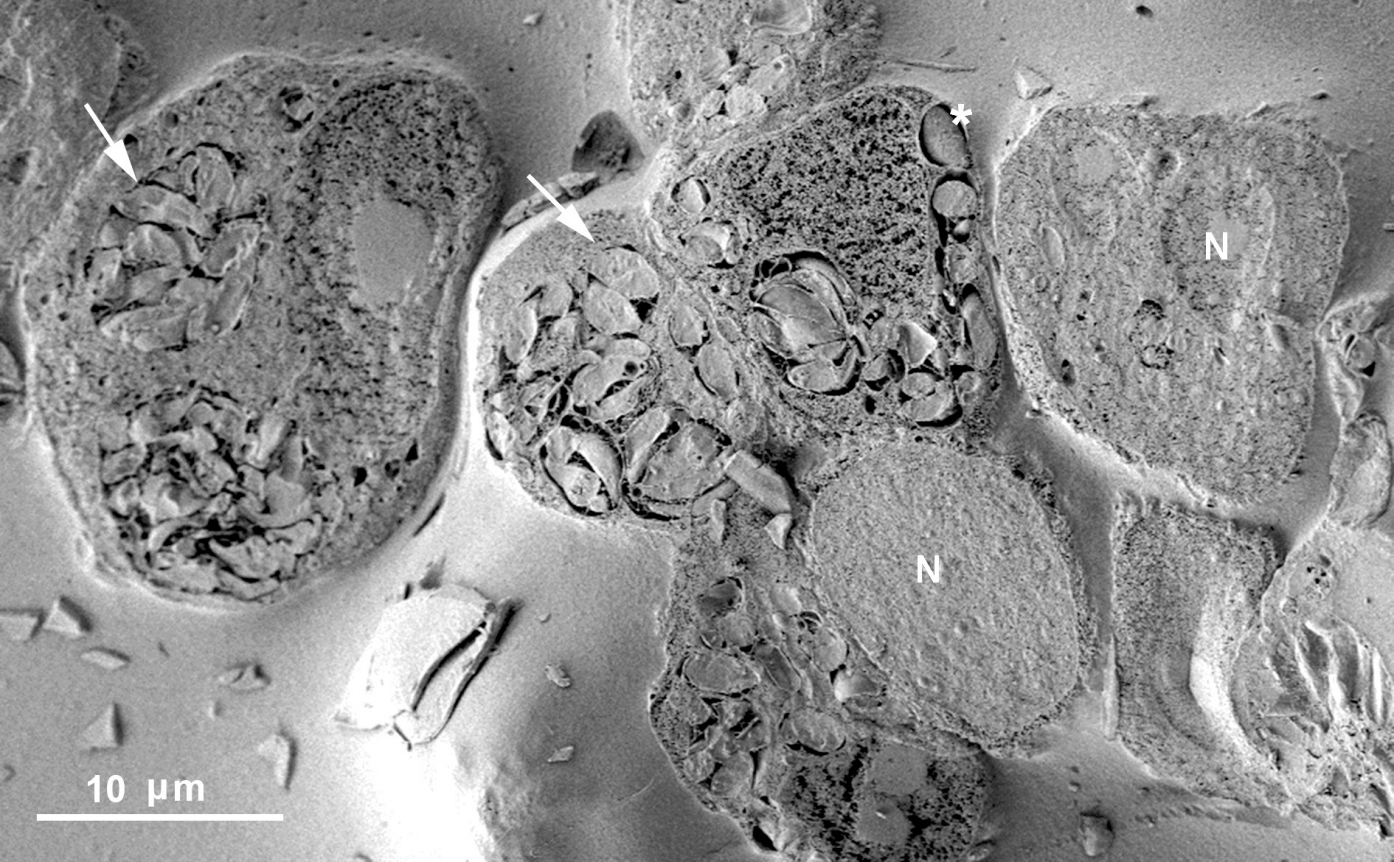
*Toxoplasma gondii* inside Rhesus monkey kidney epithelial cells (LLCMK2). Cleavage shows heavily infected cells bearing several parasitophorous vacuoles, each containing a rosette of parasites (arrows). Some vacuoles contain a single parasite (asterisk). N; nucleus of the host cell.

**Fig. 2:**
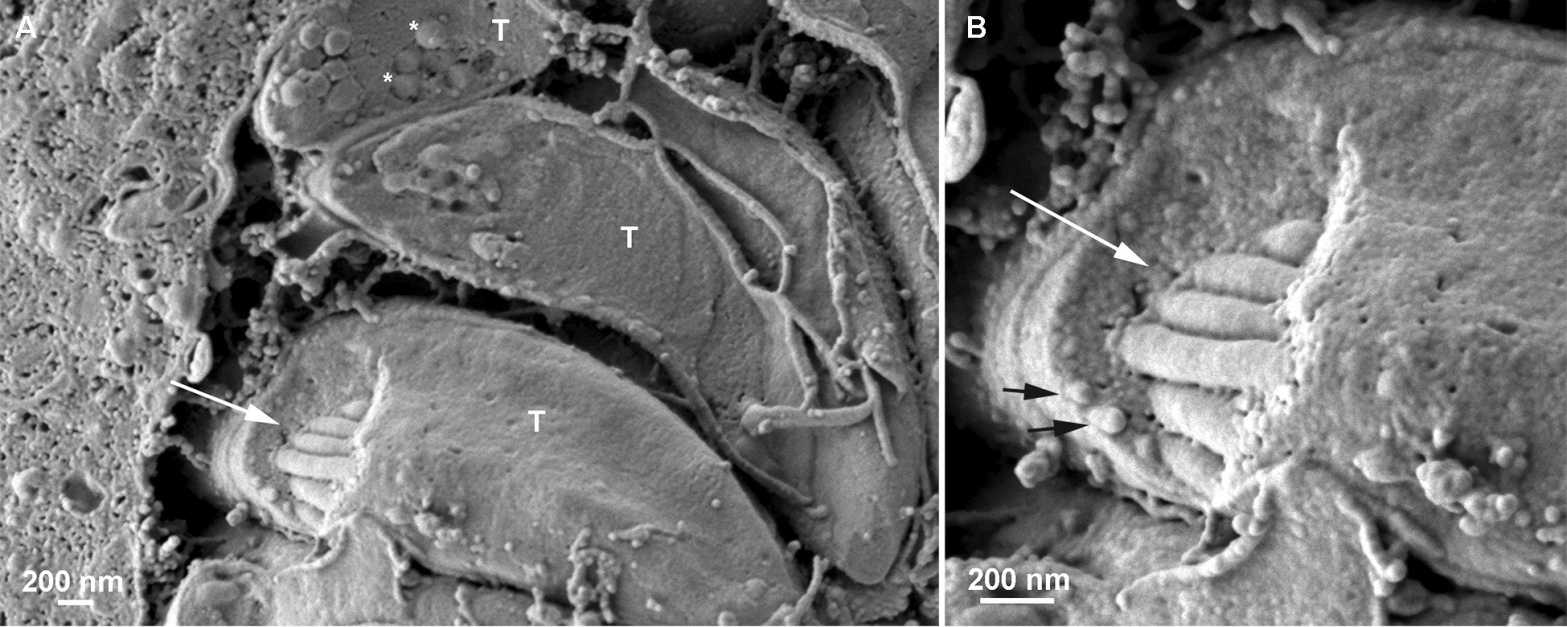
*Toxoplasma gondii* tachyzoites (T) inside a parasitophorous vacuole after cleavage. (A) In one of the tachyzoites (arrow), six rhoptries were exposed by the fracture. Asterisks indicate dense bodies; (B) higher magnification of the rhoptries (white arrow). Micronemes can also be observed under the pellicle (indicated by black arrows).

Using the present method, we noted that a random cleavage of cells infected with *T. gondii* revealed progressive levels of the internal organisation of the host cell as a whole and the distribution of the PVs inside it (Fig. 1). In each vacuole, the parasite rosettes could be complete, or the parasites could be cleaved, exposing internal organelles, as rhoptries and dense granules (Fig. 2). In freeze fracture-deep etching replicas, rhoptries could also be seen; however, the area of these replicas is much smaller, making the present protocol very useful in building a more integrated picture of the interrelation between the parasite and its host cell.^18^ Components of the intravacuolar network of tubules can be identified.

*Trypanosoma cruzi* - *T. cruzi* is a protozoan that causes Chagas disease, a condition recognised by the World Health Organisation as a neglected tropical disease. The disease impacts approximately 7 million people worldwide, with around 75 million at risk, and leads to about 12,000 deaths each year. The current chemotherapy, which includes Benznidazole and Nifurtimox, is hindered by challenges such as low specificity, high toxicity, and the emergence of drug resistance.^19^ *T. cruzi* possesses several unique cellular structures critical to its survival and pathogenicity. Among these are the kinetoplast, a dense network of mitochondrial DNA, the flagellar pocket (FP), and in some developmental forms, a cytostome, which serves as the primary site for endocytosis and exocytosis (in the case of FP), the contractile vacuole complex (CVC) that regulates osmotic pressure as well as specialised ion containing organelles such as the acidocalcisomes. *T. cruzi* also bears an elaborate cytoskeleton featuring an exuberant set of subpellicular microtubules. These specialised structures enable the parasite to adapt to various environments within the hosts and represent potential targets for therapeutic intervention. A deeper understanding of the basic cell biology of this parasite is, therefore, essential for identifying new biochemical targets, which could pave the way for the development of safer and more effective treatments.

**Fig. 3:**
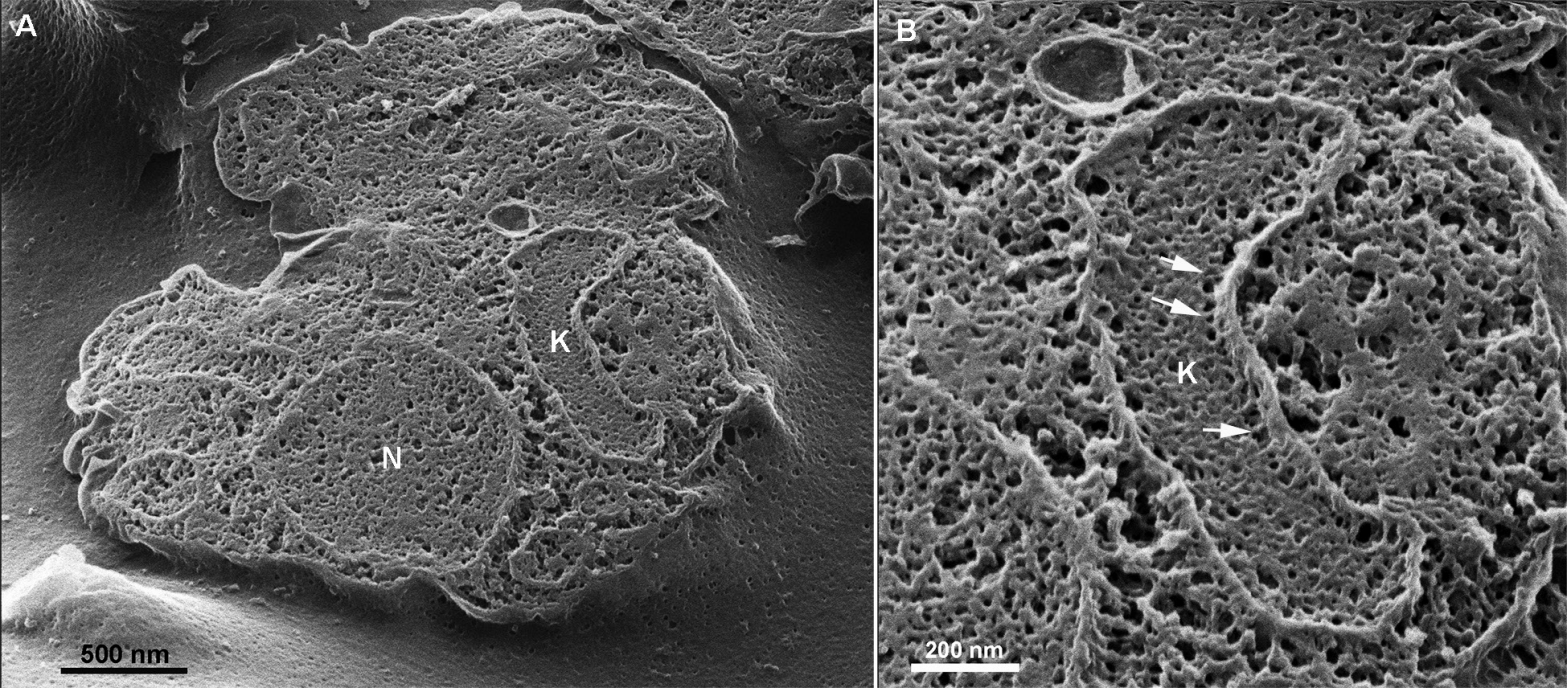
epimastigotes of *Trypanosoma cruzi* were cleaved and observed with UHR-Helium Ion Microscopy without coating or maceration. (A) General view; (B) a kinetoplast in higher magnification shows a complex network of structures in its central portion and peripheral short filaments connecting the kinetoplast to the mitochondrial membrane (arrows). N: nucleus; K: kinetoplast.

As mentioned above, once submitted to a successful cleavage, cells can potentially be analysed by any SEM. To better understand the potential of combining these techniques with an ultra-high-resolution imaging method that does not require sample coating, *T. cruzi* epimastigotes (free parasites in suspension) and *T. cruzi*-infected host cells were fractured and analysed using conventional SEM or Helium Ion Microscopy. The resulting images depicted a fair description of *T. cruzi* structures, such as the nucleus, which appears as a large, irregularly shaped structure with a relatively dense and textured appearance, and the kinetoplast, a specialised region containing the mitochondrial DNA. The kinetoplast is visible as a dense, oval-shaped structure displaying a fine network of short interconnected filaments in its central portion, most probably composed of DNA minicircles, that is connected via short filaments to the inner portion of the mitochondrion (Fig. 3).

The surrounding areas in both structures are filled with a porous, network-like matrix that likely represents other intracellular elements that were not completely exposed during sample preparation. Nevertheless, the results show the potential of Helium Ion Microscopy to provide high-resolution, detailed views of the intracellular milieu in these parasites.

**Fig. 4:**
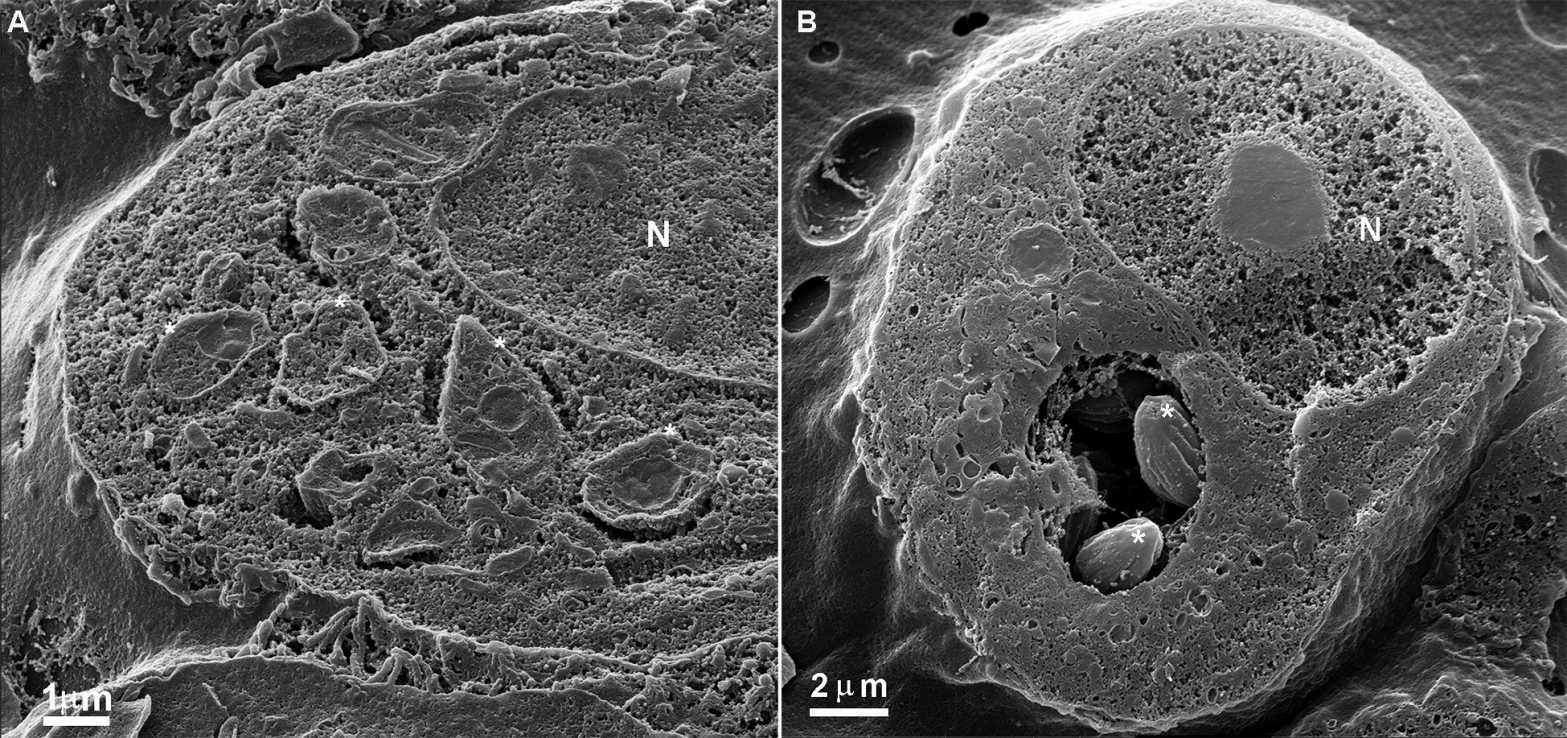
a host cell (Rhesus monkey kidney epithelial cells - LLCMK2) was infected with *Trypanosoma cruzi* using the cleavage technique without maceration. The cells were trypsinised, fixed, cleaved, coated with gold (Au), and observed in an ion beam scanning microscope (SM). (A) Parasites (asterisks) are observed in the host cell’s cytoplasm; (B) two amastigotes are observed inside a parasitophorous vacuole. N: nucleus.

Host cells infected with *T. cruzi* and cleaved using this method showed intracellular epimastigotes and amastigotes, as is usually observed by fluorescence, differential interference microscopy (DIC), or phase contrast microscopy. Parasites developing within the parasitophorous vacuole or in cytoplasmic regions, depending on the cycle stage, were easily recognised (Fig. 4). The increased depth of focus allowed the simultaneous in-focus visualisation of different parasites and host cell regions (*i.e.*, the external surface), highlighting this technique’s potential to capture multiple cellular events at varying depths.

**Fig. 5:**
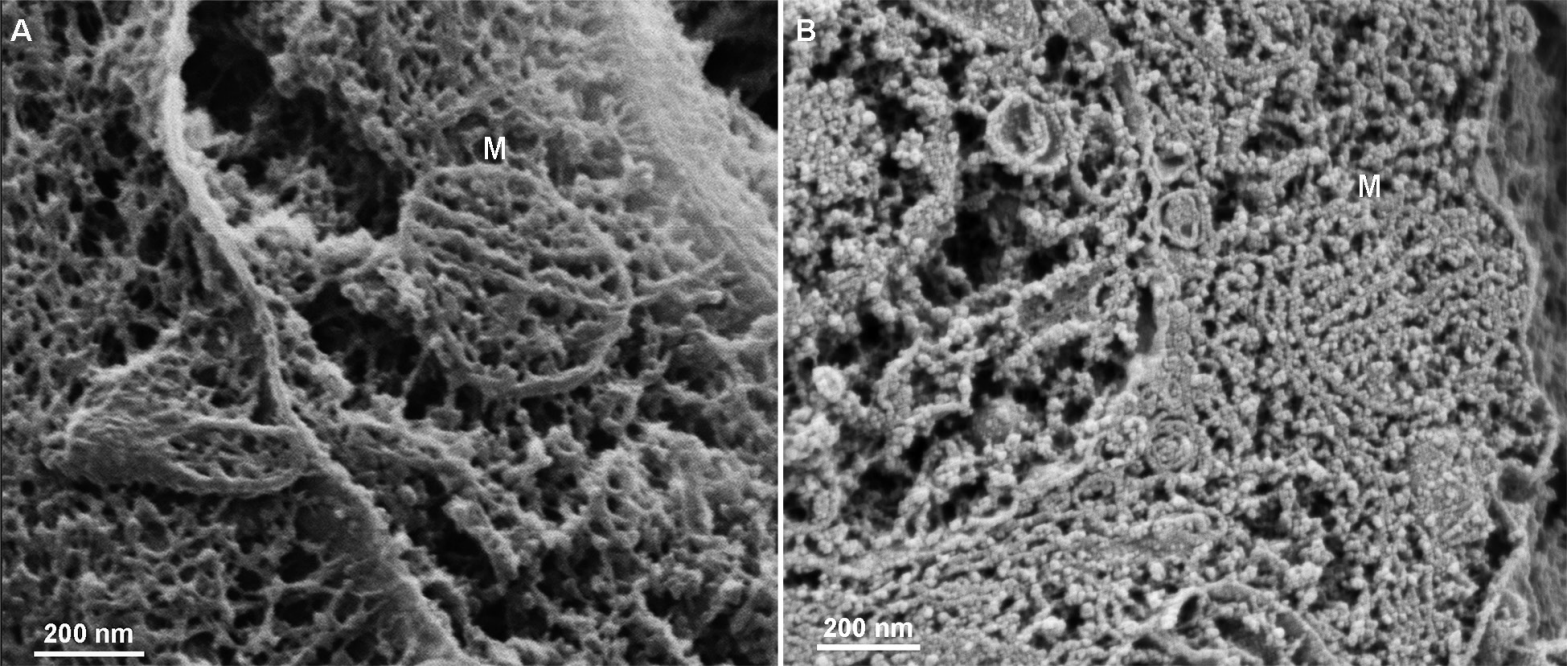
macrophage cell line (RAW 264.7 from ATCC) after 120 h of maceration. Mitochondria (M) were observed after the extraction of the cytoplasm. The images were obtained with an ion beam scanning electron microscopy (SEM) without conductive coating (A) and under a high-resolution SEM Auriga with a 5 nm thick platinum coating (B).

To verify whether this technique can be applied to non-parasitic cells, we attempted to use established cell lines where the presence of macrophages (Fig. 5) is well established. Our results demonstrated the effectiveness of the methodology in non-parasitic cells, as mitochondria were visualised without apparent distortions, even without conductive coating, and also in a high-resolution SEM Auriga with a 5 nm thick platinum coating.

*Giardia intestinalis* - *G. intestinalis* is the etiological agent of Giardiasis, an intestinal illness that affects a broad spectrum of vertebrates, including humans. An individual infected with the parasite may experience symptoms such as diarrhoea, flatulence, abdominal pain, nausea, and vomiting, although others may not develop clinical manifestations.^20^ Following this, recent studies also suggest an association between Giardiasis and the subsequent development of irritable bowel syndrome in the later post-infection period.^21^ ^,^ ^22^ Special attention should be given to infected children, as the clinical condition can worsen and cause physical growth retardation, intellectual delay, and severe malnutrition. The life cycle of Giardia is monoxenic, characterised by the presence of two morphologically distinct forms: the oval, immobile cyst, which is the infective form, and the elongated, motile trophozoite, which parasitises the host’s small intestine. Infection of the individual occurs through the ingestion of cysts in contaminated water and food, or via the faecal-oral route.^23^ Due to the ease of transmission through water, Giardiasis is closely associated with areas with inadequate sanitary conditions.

*Giardia* trophozoites are responsible for establishing parasitism; they proliferate and adhere to the epithelium of the duodenum and jejunum, forming a monolayer that causes local inflammation and effectively initiates the giardiasis condition. The parasite exhibits peculiar features, including two nuclei and unusual organelles, such as mitosomes and peripheral vesicles.^24^ ^,^ ^25^ The elaborated cytoskeleton includes microtubular structures such as the ventral disc, the median body, the funis, and the four pairs of flagella, which are crucial for biological processes such as motility, adhesion, cell division, and differentiation.^26^ ^,^ ^27^ ^,^ ^28^ ^,^ ^29^ ^,^ ^30^ Although it participates in important processes, questions remain open regarding the organisation and composition of these cytoskeletal elements.

**Fig. 6:**
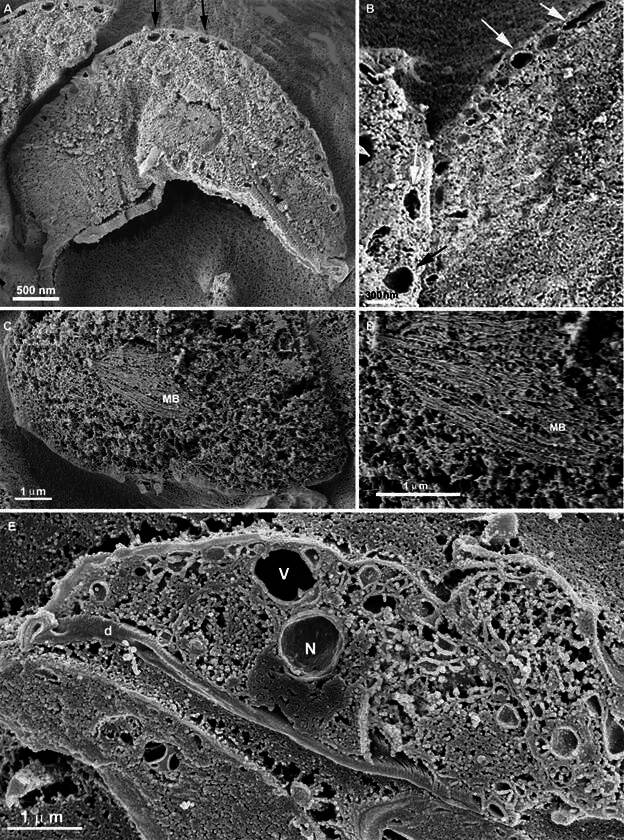
*Giardia intestinalis* was observed after fracture and maceration. (A-B) Notice the parasite’s general morphology. Arrows point to peripheral vesicles; (C-D) median body and its microtubules (MT); (E) general parasite view showing the fractured ventral disc (d); nucleus (N), and one large vacuole (V).

**Fig. 7:**
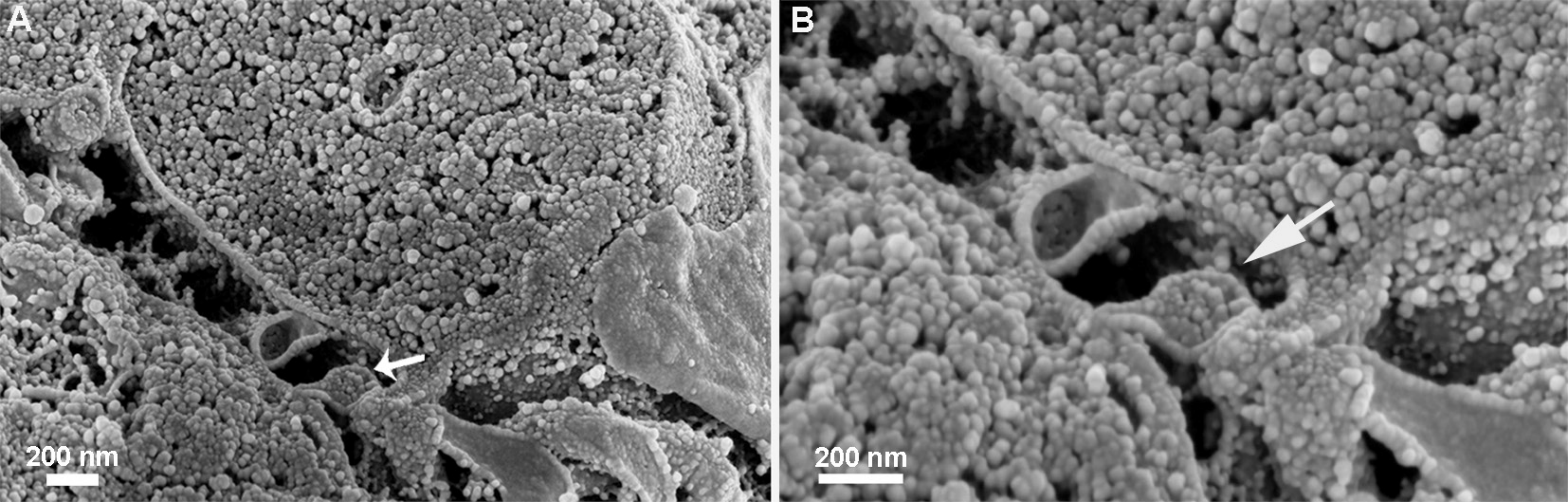
*Giardia intestinalis* was observed after fracture and maceration using HR-scanning electron microscopy (HR-SEM). (A) Fracture of the anterior region (arrow); (B) details are in high magnification of the intracellular portion of the axoneme and its externalisation region. The arrow points to the exit area of the flagellum; (C-D) low and high magnification of the fractured ventral region. The arrow points to the normal morphology of the ventral flagellum.

The standardisation of protocols for observing parasitic protozoa, combined with recent advances in bioimaging technologies, has led to a more detailed understanding of the organisation of previously known structures and the description of new arrangements and elements associated with them.^28^ ^,^ ^31^ Here, we employed a combination of hand freeze-fracture and osmium maceration, which has proven effective in studying the ultrastructure of *G. intestinalis* and provides a more comprehensive view of its cellular morphology. Random fracture of *G. intestinalis* trophozoites, observed by high-resolution scanning microscopy, allowed visualisation of the shape and distribution of organelles, such as peripheral vesicles (Fig. 6A). The cytoskeleton was also visible. It was possible to identify the microtubules of the median body and the fascicles that compose this structure (Fig. 6B), as previously described by Piva and Benchimol.^32^ Vacuoles containing concentric membranes similar to autophagic vacuoles were also defined (Fig. 6C). The region of the emergence of the flagellum (Figs. 7A-B), where an extra-axonemal structure was recently characterised,^31^ was also seen. The fin-like protrusion of the ventral flagella was visualised and confirmed through this method (Fig. 7C-D). The preservation of morphology suggests that the combined freeze-fracture and osmium maceration technique is minimally disruptive. Instead, it maintains structural integrity, thus accurately representing the trophozoite’s nanoarchitecture. The clear structural views afforded by this technique suggest its potential application in studying the details of the cytoskeleton and other subcellular structures in this organism.

**Fig. 8:**
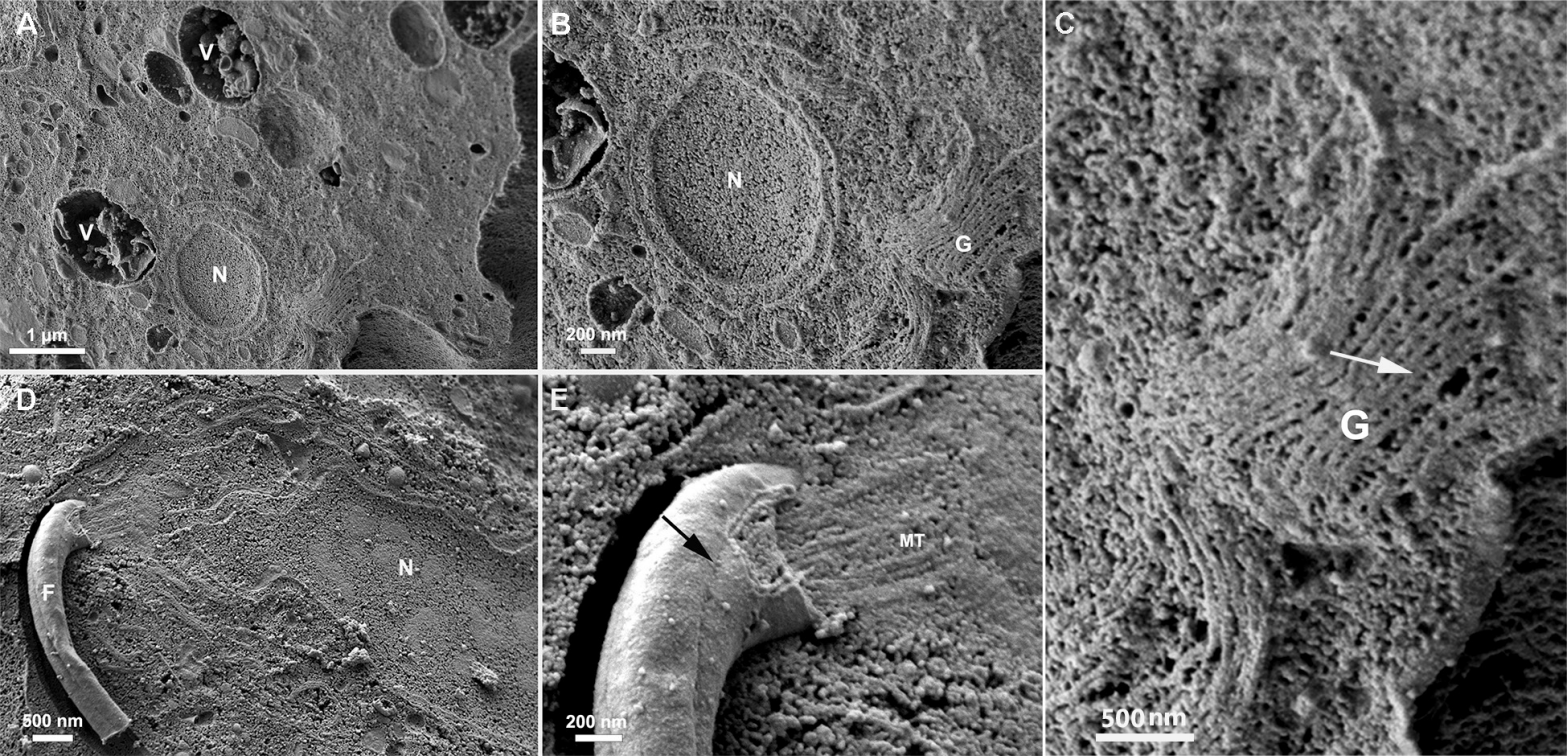
scanning electron microscopy (SEM) images of the methodology involving cleavage and maceration with osmium tetroxide in *Trichomonas vaginalis*. (A) The cleavage plane shows vacuoles (V) containing materials incorporated by endocytic activity, as well as the nucleus (N); (B) higher magnification shows the nucleus and the Golgi (G); (C) Golgi (G): the arrow points to filaments connecting the cisternae. This inset is a digital magnification of figure (B); (D-E) the fracture exhibits one flagellum (F), a nucleus (N), and several cell structures. The black arrow (in E) indicates the flagella externalisation and the flagellar membrane fracture. Notice the axoneme microtubules (MT).

**Fig. 9:**
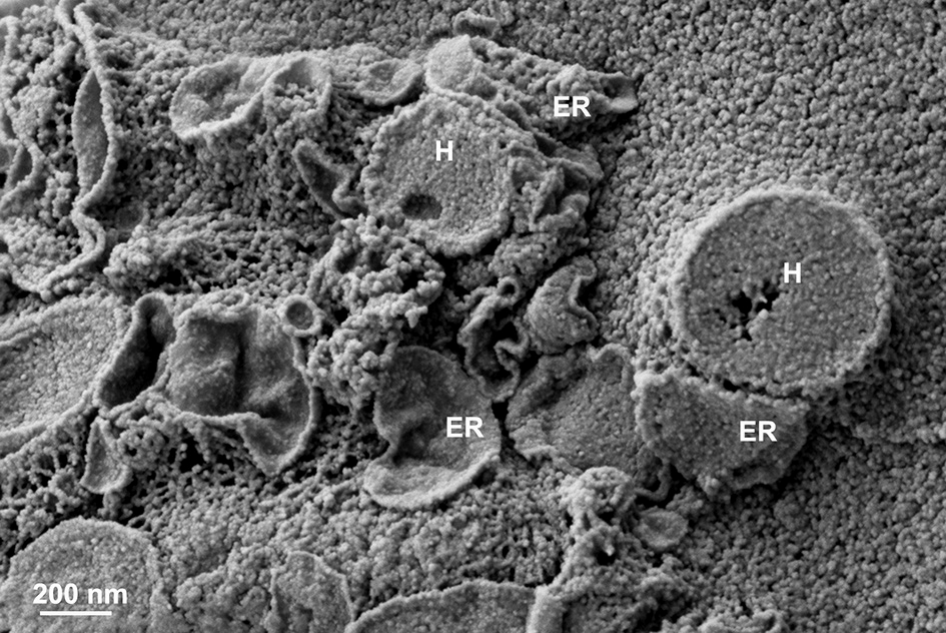
scanning electron microscopy (SEM) images of the methodology involving cleavage and maceration with osmium tetroxide in *Trichomonas vaginalis*. (A) The cleavage plane shows the cytosol, the hydrogenosomes (H), and the membranous profiles of the endoplasmic reticulum (ER). Notice the proximity of the ER to the hydrogenosomes.

*Trichomonas vaginalis* - *T. vaginalis* is the protozoan responsible for trichomoniasis, the most common non-viral sexually transmitted infection.^33^ This condition can lead to reproductive tract inflammation, adverse pregnancy outcomes, and an increased risk of HIV, other viral infections, and reproductive system cancers.^34^ This extracellular protozoan features typical eukaryotic organelles, including the nucleus, endoplasmic reticulum, lysosomes, and Golgi complex (Fig. 8). However, it is amitochondrial and contains hydrogenosomes (Fig. 9), which generate molecular hydrogen and adenosine tri-phosphate (ATP) by oxidising pyruvate or maleate under anaerobic conditions.^35^ ^,^ ^36^ ^,^ ^37^ Endocytosis in *T. vaginalis* is essential for nutrient uptake and immune defence, with energy for this process primarily derived from glycolysis.^38^ ^,^ ^39^ ^,^ ^40^ ^,^ ^41^

Additionally, *T. vaginalis* possesses a complex and elaborate cytoskeleton called the mastigont system.^35^ ^,^ ^42^ This system includes, in addition to the four anterior flagella and one recurrent flagellum, stable microtubule-based structures such as the axostyle, which extends along the length of the cell and serves as its central axis, and the pelta, which reinforces the canal from which the flagella emerge.^42^ ^,^ ^43^ It also features striated filaments, such as the costa, a periodically striated structure that provides mechanical support and is associated with the hydrogenosomes,^35^ ^,^ ^44^ and the four parabasal filaments, which are distinct from each other.^45^ The initial step in host colonisation involves the parasite’s adherence to urogenital epithelial cells, with the cytoskeleton playing a crucial role in this process.

Concerning *T. vaginalis,* the osmium maceration technique enabled the visualisation of several cell structures. The vacuoles and their contents were well-preserved structures, presenting singular images that had not been seen before by other procedures (Fig. 8A), facilitating discussions on the endocytosis process of *T. vaginalis*. The Golgi cisternae were visible (Fig. 8B). In trichomonads, the Golgi complex interacts with two parabasal filaments on the cis face and another two on the trans face.^45^ The Golgi in trichomonads is well-developed, and the stacks of cisternae present dilations at the distal end, forming vesicles similar to those observed using the traditional freeze-fracture technique described by Benchimol and de Souza.^46^ The hydrogenosomes and their proximity to the endoplasmic reticulum can be well visualised with the maceration technique (Fig. 9). Previous studies have highlighted this proximity.^47^

The overall organisation of the membrane-bound organelles, including the nucleus, was preserved, and different regions with varying densities were observed within the nucleus (Fig. 8C). Additionally, this technique allowed for the observation of the structural organisation of the anterior region of the protozoan and the layers of the plasma membrane in the partially fractured flagellum. This same region allowed the visualisation of the flagellum’s externalised portion and the cytoplasmic portion, exposing the microtubules longitudinally (Fig. 8C-D). The fractured flagellum region exhibited the microtubules that form the axonemes (Fig. 8D). The flagellum externalisation portion is particularly interesting because it helps to understand the elements associated with the parasite’s mobility, which is important for comprehending the adaptations of this protozoan and the host-parasite interactions.

The structural integrity and high resolution of intracellular and extracellular components, afforded by freeze-fracture and osmium maceration, make it a powerful tool in cellular and microbiological research.

We noted that the present freeze-fracture and osmium maceration technique has many advantages over traditional freeze-fracture. It eliminates several steps of the traditional freeze-fracture technique. For example, (1) it does not use specialised, expensive, and sophisticated working equipment; (2) there is no need for replica creation, a step that requires expertise and is challenging for beginners since, during handling, they can be curled leading to the loss of all work; (3) the samples are analysed in a SEM and not in a TEM which is much more expensive. In addition, this method is cost-effective and significantly faster, accelerating the achievement of results and providing greater efficiency and ease of observation across different cell types.

Therefore, it presents itself as a good option for groups that lack access to laboratories equipped with expensive equipment and limited funding to acquire large volumes of cryogens. In the traditional method, dozens of litres of liquid nitrogen are used, making routine acquisition a significant challenge.

Although artefacts may be a limitation, this problem can occur with any methodology that utilises fixation and fracture. To overcome this issue, it is necessary to possess extensive knowledge of the cellular model in conventional techniques and to consult specialised literature to accurately interpret the results, thereby facilitating advances in understanding the cellular model.

Thus, the present osmium maceration technique may serve as an additional and valuable tool among the various cell biology methods and, therefore, be an economical solution for visualising cellular and subcellular structures. In addition, this method revealed some details of the relationships between cellular constituents, which could be more clearly seen in TEM preparations, as thin sections do not provide a 3D view.

Following several protocol trials, we found that even in experiments without maceration, the fractured images provided clear images of the cellular architecture of all parasite protists analysed here. Similarly, information obtained with high resolution-SEM (HR-SEM) and/or conventional SEM yielded nearly identical observations. Thus, using this technique with an expensive HR-SEM is not mandatory.

When fracturing frozen biological samples, one can get views of transverse fractures and deep views in different fracture planes of cellular structures and organelles. It allows views that have never been seen in ultrathin sections obtained for conventional TEM. This information would be directly accessible for 3D analyses and quantitative studies of the distribution of components within the cells and membranes.

Another aspect that favours the use of this technique is the large number of samples that can be analysed in a single experiment. While conventional freeze-fracture yields a maximum of three very small replicates (with luck), this technique, due to the mechanical and visual fragmentation, can achieve dozens of fragments that can be analysed, and the best results selected. On the other hand, this technique sometimes presents other difficulties, such as insufficient maceration and the presence of organic material residues. However, this problem can also occur with conventional freeze-fracture. Another problem is that the force used to break the frozen samples may not be sufficient, and the cells may remain intact, allowing only the plasma membrane to be visualised. In this case, the researcher must repeat the experiment; however, the costs will be negligible compared to those of repeating a conventional freeze-fracture. Additionally, some fragments may not contain cells, a fact resulting from the lack of concentration of the pellet in free cells such as protozoa.

Regarding possible artefacts, we sometimes observed excessive maceration, resulting in empty spaces within the cells, devoid of fibres or even cytoplasm. On the other hand, three-dimensional images that were previously rarely observed, such as the extra-axonemal structure in *G. intestinalis* flagella,^31^ were successfully captured with this methodology. Additionally, a large number of cells are obtained, with different fragmentation points, which allows for a careful and statistically relevant analysis of dubious information. The large number of fragments obtained is also a major advantage of conventional freeze-fracture, including the possibility of not using metal coverings, which in conventional freeze-fracture could often mask fine details requiring high resolution. On the other hand, the ease of repeating experiments on the same day with virtually no cryogenic costs makes this methodology a major asset for advancing knowledge of the cell biology of various cell models.

This technique reveals enormous potential in analysing multiple cells, providing meaningful insights into cell biology. However, slight adaptations to chitosan concentration and maceration times must be attempted for each cell type. Additionally, as with any method, it can produce specific artefacts that must be recognised and understood, such as empty spaces due to excessive maceration.

In conclusion, this updated application of a classic technique enables the analysis of various cells and tissues at the ultrastructural level in a cost-effective manner, allowing laboratories with limited resources to investigate their cell models and contribute to the understanding of cellular architecture [Supplementary data (Figure)].

## Supplementary Materials

Supplementary material
