# Supplementary material for: Breaking the ice − simplified freeze-fracture of parasitic protists: a cost-effective approach without highly specialised equipment
Source: Mem Inst Oswaldo Cruz. 2025 Dec 15;120:e250060. doi: 10.1590/0074-02760250060 (PMC12711207; doi:10.1590/0074-02760250060)
Supplement: Supplementary material [file 1678-8060-mioc-120-e250060-s.pdf]

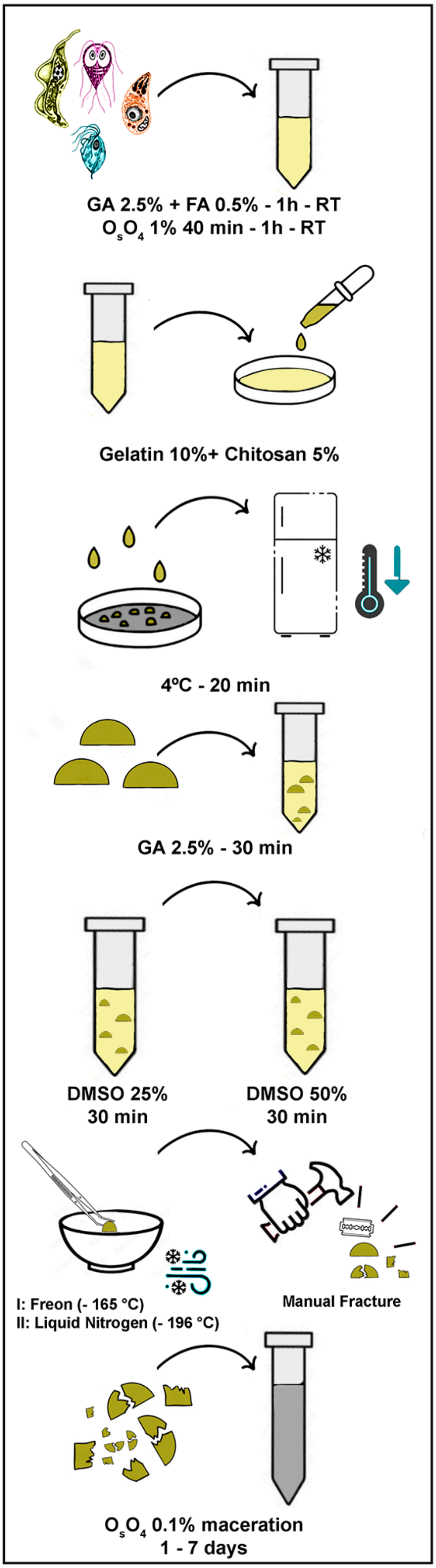

Scheme showing the steps of cell cleavage. The protists are fixed, embedded in gelatine and chitosan, and placed in the refrigerator. They are cryoprotected with dimethyl sulfoxide (DMSO) and frozen in Freon and liquid nitrogen. The fracture is performed using a pre-cooled razor blade or hammer, and daily changes of 0.1% OsO_4 carry out maceration.
